# Cytosolic phosphorylated EGFR is predictive of recurrence in early stage penile cancer patients: a retropective study

**DOI:** 10.1186/1479-5876-11-161

**Published:** 2013-07-02

**Authors:** Giuseppe Di Lorenzo, Sisto Perdonà, Carlo Buonerba, Guru Sonpavde, Vincenzo Gigantino, Giuseppe Pannone, Giuseppe Quarto, Matteo Ferro, Gabriella Gaudioso, Daniela Terracciano, Rossella Di Trolio, Pasquale Rescigno, Gerardo Botti, Sabino De Placido, Gaetano Facchini, Paolo A Ascierto, Renato Franco

**Affiliations:** 1 Genitourinary Cancer Section and Rare-Cancer Center, Medical Oncology Division, University Federico II, Napoli, Italy; 2 Urology Division, ISTITUTO NAZIONALE TUMORI "FONDAZIONE G. PASCALE"-IRCCS, Naples, Italy; 3 University of Alabama at Birmingham Comprehensive Cancer Center, Birmingham, AL, USA; 4 Pathology Unit, ISTITUTO NAZIONALE TUMORI "FONDAZIONE G. PASCALE"- IRCCS, Naples, Italy; 5 Department of Surgical Sciences; Section of Anatomic Pathology; Second Section of Oral Pathology, University of Foggia, Foggia, Italy; 6 Division of Urology, European Institute of Oncology, Milan, Italy; 7 Unit of Medical Oncology and Innovative Therapy, Department of Melanoma, Sarcoma, and Head and Neck Cancers, ISTITUTO NAZIONALE TUMORI "FONDAZIONE G. PASCALE"- IRCCS, Naples, Italy; 8 Division of Medical Oncology, Department of Urology and Gynecology, ISTITUTO NAZIONALE TUMORI "FONDAZIONE G. PASCALE"- IRCCS, Naples, Italy

**Keywords:** Epidermal growth factor receptor (EGFR) expression, Phospo-EGFR, Immunohistochemistry, Penile cancer, Tissue micro-array (TMA)

## Abstract

**Background:**

Penile cancer (PC) is a rare tumor, and therapeutic options are limited for this disease, with an overall 5-year overall survival around 65-70%. Adjuvant therapy is not recommended for patients with N0-1 disease, despite up to 60% of these patients will die within 5 years from diagnosis.

**Methods:**

Medical records of all patients who underwent radical surgery at University Federico II of Naples and at National Tumor Institute “Pascale” of Naples for early squamous cell carcinoma of the penis from January, 2000 to December, 2011 were retrieved. Paraffin wax embedded tissue specimens were retrieved from the pathology archives of the participating Institutions for all patients. Expression of p-EGFR, EGFR and positivity to HPV were evaluated along with other histological variables of interest. Demographic data of eligible patients were retrieved along with clinical characteristics such as type of surgical operation, time of follow up, time of recurrence, overall survival. A multivariable model was constructed using a forward stepwise selection procedure.

**Results:**

Thirty eligible patients were identified. All patients were positive for EGFR by immunohistochemistry, while 13 and 16 were respectively positive for nuclear and cytosolic p-EGFR. No EGFR amplification was detected by FISH. Eight patients were positive for high-risk HPV by ISH. On univariable analysis, corpora cavernosa infiltration (OR 7.8; 95% CI = 0,8 to 75,6; P = 0,039) and positivity for cytosolic p-EGFR (OR 7.6; 95% CI =1.49 to 50; P = 0.009) were predictive for recurrence, while only positivity for cytosolic p-EGFR (HR =9.0; 95% CI 1.0-100; P = 0,0116) was prognostic for poor survival.

**Conclusion:**

It is of primary importance to identify patients with N0-1 disease who are at increased risk of recurrence, as they do not normally receive any adjuvant therapy. Expression of p-EGFR was found in this series to be strongly related to increase risk of recurrence and shorter overall survival. This finding is consistent with the role of p-EGFR in other solid malignancies. Integration of p-EGFR with classic prognostic factors and other histology markers should be pursued to establish optimal adjuvant therapy for N0-1 PC patients.

## Introduction

Incidence of penile cancer (PC) is around 0,5-1 per 100000 males in Europe and in the United States, climbs to 8.3 per 100 000 in parts of Asia, Africa and South America [1]. Squamous cell carcinoma represents the greatest majority of PC cases [1]. Therapeutic options are limited for this disease, with a 5-year overall survival around 65-70%, and no significant improvement over the past two decades [2]. Prognosis is dismal in patients not amenable to radical surgery, who generally survive less than 12 months [3]. After surgical removal of the primary tumor, patients with clinically positive nodes should undergo regional lymphadenectomy, while in patients with clinically negative lymphnodes, nomograms and sentinel-node biopsy are extremely useful to advice for lymphadenectomy [4].

Adjuvant chemotherapy is recommended in patients with N2 or N3 lymphnode involvement [4]. Although node status is the major prognostic determinant in PC, a significant number of clinical and biological factors of prognostic value have been identified. A nomogram, including growth pattern, histologic grade, lymphatic or venous embolization, corpora cavernosa and spongiosum infiltration and pathological regional lymph node involvement showed a concordance index of around 74% for cancer specific 5-year survival [5]. A number of biological variables have also proved to have a potential prognostic role in PC, including HPV [6], periostin [7], p53 [8], such as ki67, mini-chromosome maintenance 2 protein (MCM2) and geminin [9].

The epidermal growth factor receptor is a member of the HER/erbB family of receptor tyrosine kinases, which also includes HER2 (neu, erbB2), HER3 (erbB3), and HER4 (erbB4) [10]. Ligand binding and dimerization of EGFR causes autophosphorylation of the intracytoplasmic domains and activation of the intracellular tyrosine kinase. EGFR is overexpressed in the majority of PC patients [11]. Nevertheless, in a recent work conducted in 148 PC tissue samples, the phosphorylated active form of EGFR was detected in only a fourth of all samples [10]. EGFR phosphorylation appeared to be predictive of poor prognosis in a number of malignancies, including non-small-cell lung cancer, breast cancer and oropharyngeal cancer [12-14].

The aim of this study was to gain further insight into the prognostic role of p-EGFR status in early stage PC patients, which was assessed in a sample of thirty men after controlling for known prognostic factor in PC using a multivariable model.

## Patients and methods

### Patients

Medical records of all patients who underwent radical surgery at University Federico II of Naples and at National Tumor Institute “Pascale” of Naples for early squamous cell carcinoma of the penis from January, 2000 to December 2011 were retrieved. Patients were required to have assessment of inguinal lymphnode status via sentinel node biopsy or lymphadenectomy. Patients with > N1 or M1 disease or those who received perioperative chemotherapy were excluded from this analysis. Demographic data of eligible patients were retrieved along with clinical characteristics such as type of surgical operation, time of follow up, time of recurrence, overall survival. Local research ethics committee approval for the study was obtained.

### Histology review

Paraffin wax embedded tissue specimens were retrieved from the pathology archives of the participating Institutions for all patients. The lack of fresh tissue specimens did not affect the quality of the analyses performed. Excised tumors were histologically staged using the revised TMN system criteria 2002 [15]. Pathological variables of the primary tumor included: grade, local stage, subtype, and lymphovascular invasion. Tumor grade was defined using Broders's classification [16]. Lymphovascular invasion was determined microscopically and confirmed using antibodies against endothelial markers CD33 and CD34. Lymph node status at the time of diagnosis was confirmed following pathological review of sentinel node biopsy and inguinal nodes specimens obtained with lymphadenectomy.

### TMA building

A Tissue Micro-Array (TMA), has been built using 35 tissue samples, including 30 penile squamous cell carcinoma (PSCC) and 5 normal tissues. A single block for each case has been selected for the TMA building, identifying the most representative tumoral areas from blocks of surgical samples as well as normal gland tissue where possible. All tumours and controls have been reviewed by two experienced pathologists.

Tissue cylinders with a diameter of 1 mm have been punched from morphologically representative tissue areas of each ‘donor’ tissue block and brought into one recipient paraffin block (3 × 2.5 cm) using a semiautomated tissue arrayer , CK 3500 Tissue Microarrayer; ISE TMA Software (Galileo TMA, Integrated System Engineering, Milan, Italy).

### Immunohistochemistry analysis

Immunohistochemical analysis has been performed on 4-μm TMA serial sections from formalin-fixed, paraffin embedded tissues, in order to evaluate the expression of EGFR (clone 3C6 EGFR Ventana); p-EGFR Tyr 845 (clone 2231 phospho-EGF receptor (Tyr845) Cell signaling). Negative control slides without primary antibody were included for each staining.

Paraffin slides have been deparaffinized in xylene and rehydrated through graded alchols. Antigen retrieval has been performed with slides heated in 1 mM EDTA buffer, pH 8.0 in a bath for 20 min at 97°C. After antigen retrieval, the slides allow to cool. The slides have been rinsed with TBS and the endogenous peroxidase has inactivated with 3% hydrogen peroxide. After protein block (BSA 5% in PBS 1x), every slides have been incubated with specific primary antibody: anti phospho-EGFR tyr845 (rabbit 1:100 over night). The sections have been rinsed in TBS and incubated for 20 minutes with Novocastra Biotinylated Secondary Antibody (RE7103), a biotin-coniugated secondary antibody formulation that recognized mouse and rabbit immunoglobulins. Then the sections have been rinsed in TBS and incubated for 20 minutes with Novocastra Streptavidin-HRP (RE7104) and then peroxidase reactivity has been visualized using a 3,3’-diaminobenzidine (DAB). Finally, the sections have been counterstained with hematoxylin and mounted. Results are interpreted using a light microscope.

The results of the immunohistochemical staining were evaluated separately by two observers. In each tissue section 10 representative high power fields (HPFs) were analyzed at optical microscope (OLYMPUS BX41, at x40) and were selected for EGFR positive tumor cells with an average of 1000 tumor cell per case and 200 tumor cells per field. The topographical staining pattern was also evaluated and recorded as membranous (M), cytoplasmic (C), or mixed, and nuclear (N). For each case, the cumulative percentage of positive cells among all sections examined was determined as follows: 0 points for negative staining of the considered cells, (1) <10%, (2) 10-50%, (3) 51-80% and (4) ≥ 80% positive staining of the considered cells. The intensity of staining was scored as 0, no staining; +, weak; ++, moderate; +++, strong.

For p-EGFR Tyr 845 and p-EGFR Tyr 1068 immunohistochemical evaluation, we selected a dichotomized indicator variable. We established a cut-off point at 5%: the cells were considered positive when > 5% of them showed a cytoplasm staining, and negative when no staining was observed or < 95% of cells stained for the marker. Intensity of staining was not scored for  p-EGFR [14].

### FISH analysis

FISH analysis has been performed on representative sections of PSCC TMA. The slides were deparaffinized and hydrated than they were immersed in citrate buffer (ph 6) for 15 min at 85°C. The slides were then rinsed in distilled water for 5 min for two times. The slides pre-treatment and protease incubation were performed according the manufactures illustrated in datasheet of Vyses (paraffin pre-treatment reagent kit II).

The probes utilized were the commercial LSI EGFR Dual-Color Probe-Hyb Set (Vysis/Abbott Molecular, IL) LSI EGFR Spectrum Orange/Cep-7 Spectrum Green in order to simultaneously visualize EGFR gene and chromosome 7 copy number according to manufacturer’s instructions. DAPI II (4,6-diamino-2-phenyindole-2-hydrochloride) was used for chromatin counterstaining. The fluorescence signals (orange for LSI EGFR, green for Cep-7, and blue for nuclear chromatin) were evaluated under epifluorescence microscope (Olympus). Image acquisition was done by CCD microscopy camera (Olympus). Signals were evaluated by two assessors scoring at least 60 interphase nuclei in four different high power fields (HPF). The FISH results were scored as follow: specimens with the ratio LSI EGFR/CEP-7 ≥2.0 were considered as amplified; polysomic were considered cases showing three or more CEP-7 signals per cell in more than 20% of the evaluated cells.

### In situ hybridization (ISH) for HPV-DNA detection

For HPV detection has been used an HPV ISH sistem(Ventana Inform HPV, Tucson, AZ, USA) to detect integration or episomic status in our cases included on Tissue Micro Array.

The commercially available Ventana kit includes the following probes for HR-HPVs 16, 18, 31, 33, 35, 39, 45, 51, 52, 56, 58, e 66 (Iiform HPV family-III 16 Probe; Ventana - Roche); and the following probes for LR-HPVs.

### Statistical methods

Overall survival (OS) was calculated from date of diagnosis to death. Time to recurrence was calculated from date of diagnosis to recurrence. Descriptive statistics, confidence intervals [CI] and frequency counts were used to summarize characteristics of the study population. Time to recurrence and overall survival (OS) were calculated using the Kaplan-Meier method. Cox proportional hazards regression was used to investigate for prognostic factors of time to recurrence and OS, while logistic regression analysis were used to investigate for prognostic factors of recurrence at any time as a dichotomous variable. A multivariable model was constructed using a forward stepwise selection procedure. A p value < 0.05 was considered statistically significant and all tests were two-sided.

## Results

### Patient’s characteristics

All 30 patients had early stage squamous cell penile carcinoma, with the majority of them having classic squamous cell penile carcinoma. Median age at diagnosis was 58 years (53–68) and the patients were diagnosed between the years 2000 to 2011 and the median follow-up was 2 years.

All patients were positive for EGFR by IHC, while 13 and 16 were respectively positive for nuclear and cytosolic p-EGFR. No EGFR amplification was detected by FISH. Eight patients were positive for high-risk HPV by ISH. Patients’ characteristics are detailed in Table 1.

**Table 1:** Patients’ characteristics

**Characteristics**		**Sample population**
**N**	**Number (%)**	**median(range)**
Age		58 (53–68)
T stage		
T1	5	
T2	24	
T3/4	1	
Grade		
G1	9	
G2	14	
G3	7	
Sentinal node byopsy		
Yes	26	
No	4	
Lymphadenectomy		
Yes	10	
No	20	
Lymphnode status*		
N0	21	
N1	9	
Histology		
Classic	23	
Basaloid	6	
Verroucous	1	
Corpus spongiosum infiltration		
Yes	18	
No	12	
Corpora cavernosa infiltration		
Yes	7	
No	23	
Uretrha infiltration		
Yes	1	
No	29	
Lymphovascular invasion		
Yes	10	
No	20	
Surgical operation		
Local excision	3	
Glansectomy	1	
Partial penectomy	11	
Total penectomy	15	
High grade HPV status		
Positive	8	
Negative	22	
p-EGFR status		
Nuclear		
Positive	13	
Negative	17	
Cytosolic		
Positive	16	
Negative	14	
EGFR amplification status		
Amplified	0	
Aneuploid	8	
Euploid	17	
Not evaluated	5	

*As assessed by sentinal node biopsy and/or lymphadneectomy.

### Staging and surgical management

All patients were treated with radical surgery. Physical examination and biopsy for histology diagnosis were performed before surgery in all patients. All patients had either sentinel node biopsy or standard inguinal lymphadenectomy or both. Often patients undergoing inguinal lymphadenectomy, nine had N1 disease. An abdominal CT scan with and without contrast excluded pelvic lymphnode involvement in patients with N1 disease. Sentinel node biopsy was performed according to published guidelines [4].

### Recurrence and survival

**Figure 1:**
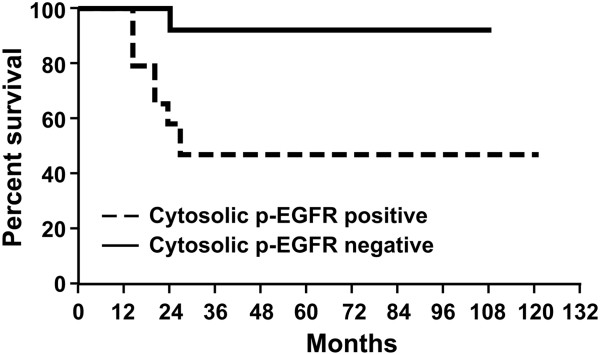
Overall survival.

All patients of the sample population had a recorded survival time. Of these, 10 patients were dead (33%) and 20 alive (66%) at the time of analysis. Penile cancer was the cause of death of eight patients. Median survival time was 25 months (20–50) (Figure 1). Sixteen patients presented recurrent disease. In these patients, median time to disease recurrence was 12.5 months (9–16.5). Grade, lymphnode status, histology, HPV status, lymphovascular invasion, cytosolic and nuclear p-EGFR status, corpora cavernosa and corpus spongiosum infiltration were assessed on univariable nad multivariable analysis as predictive of recurrence and survival. On univariable analysis, corpora cavernosa infiltration (OR 7.8; 95% CI = 0,8 to 75,6; P = 0,039) and positivity for cytosolic p-EGFR (OR 7.6; 95% CI =1.49 to 50; P = 0.009 ) were predictive for recurrence (Table 2), while only positivity for cytosolic p-EGFR (HR =9.0; 95% CI 1.0-100; P = 0,0116) was prognostic for poor survival. At multivariate analysis, only cytosolic p-EGFR was associated to increased risk of recurrence and poor survival.

**Table 2:** Logistic regression anlysis for disease recurrence

**Characteristic**	**Hazard ratio (95% CI)**	**p-value**
**Univariable**		
**Cytosolic p-EGFR (Positive vs. Negative)**	**7.6 (1.49 to 50)**	**P = 0,009**
Nuclear p-EGFR (Positive vs. Negative)	0,6 (0,1 to 2,5)	P = 0,490
**Corpora cavernosa infiltration Yes vs. no**	**7,8 (0,8 to 75,6)**	**P = 0,039**
Corpus spongiosum infiltration no vsyes	0,4 (0,1 to 2)	P = 0,294
Age	0,9 (0,9 to 1)	P = 0,807
Lymphovascular invasion Yes vs no	2,8 (0,5 to 14,3)	P = 0,190
Grade	1,7 ( 0,6 to 4,7)	P = 0,295
Histology	5,9 (0,5 to 58,4)	P = 0,086
HPV positive vs. negative	0,8 (0,1 to 4,2)	P = 0,825
Euploid vs. Aneuploid	1,2000 0,2464 to 5,8440	P = 0,821
**Multivariable**		
Cytosolic p-EGFR (Positive vs. Negative)	7.6 (1.49 to 50)	P = 0,009

## Discussion

In this retrospective analysis of 30 PSCC patients with ≤ pN1 disease undergoing surgery without perioperative chemotherapy, tumor p-EGFR was the only significant prognostic factor on multivariable analysis. Discriminating expression of phosphorylated and non phosphorylated EGFR has a biological and clinical significance in multiple human cancers, e.g. non-small cell lung cancer and breast cancer. In a retrospective study, conducted in 36 patients with non small cell lung cancer, EGFR phosphorylation, which was not associated to EGFR overexpression, was correlated to shorter time to progression and overall survival at univariate analysis [12]. Similarly, in a series of 154 women with invasive breast cancer, EGFR and pEGFR proteins were found in 11.3% and 35.7% of patients, respectively, and univariate and multivariate analysis showed that the EGFR/pEGFR phenotype was significantly associated with poor overall survival [13]. In one study of penile cancer patients, p-EGFR was expressed in 25% of 148 samples, but an independent prognostic impact was not reported. A strong positive correlation of p-EGFR with phosphorylated nuclear and cytoplasmic Akt was identified, which is consistent with activated EGFR acting as an upstream regulator of PI3K-Akt. Specifically, a strong negative correlation of p-EGFR was found with PTEN protein expression, which is explained by PTEN-mediated EGFR downregulation, and with HPV, which suggests that distinct biological pathways are activated in HPV negative tumors [10]. High-risk HPV is approximately found in 50% of penile carcinomas [17]. Similarly to head an neck squamous cell carcinomas [18], high risk HPV was found to be positively correlated to outcome in penile carcinoma. In a series of 171 patients with penile carcinoma, high-risk HPV DNA was detected in 29% of the tumors. Disease-specific 5-year survival in the high-risk HPV-negative group and high-risk HPV-positive group was 78% and 93%, respectively. At multivariate analysis, the HPV status was an independent predictor for disease-specific mortality (p = 0.01) with a hazard ratio of 0.14 (95% CI: 0.03-0.63) [6]. In our series, presence of high-risk HPV status did not significantly affect survival at multivariate analysis, and neither did any other established factors such as grade and stage, while p-EGFR was significantly related to an increased risk of recurrence and shorter overall survival time. In our selected population of patients with no or limited lymphnode involvement (N0-1), eight patients (26%) died after a median followed up of 25 months, which is concordant with 5-year cancer specific survival probabilities between 75% and 93% for the patients with stage cN0 disease, between 40% and 70% for those with stage cN1 disease reported by literature [5]. Despite a clinically significant risk of disease recurrence, adjuvant treatment is not recommended in N0-1 patients. Risk factors allowing risk stratification are therefore especially required in this setting. Phosphorylated EGFR is easily assessable and may be integrated along with other histological markers of tumor aggressiveness.

In one study conducted in 110 surgical specimens from patients with penile carcinoma, 27.2% of samples stained positive for p53. The 5-year cancer specific survival rate for the entire study cohort was 74%, and it was significantly lower in patients positive for p53 (51% vs. 84%, p = 0.003). At multivariable analysis, p53 showed to be strongly related to decrease survival (HR = 3.20; p = 0.041) [8]. In a similar fashion, EGFR appeared to be independently predictive of enhanced risk of recurrence and decreased survival in our series. We chose to investigate the role of EGFR in a specific and homogeneous subset of PC patients – those with N0-1 disease-, who are normally not candidates to adjuvant therapy, but at a clinically relevant risk of recurrence. Limitations of our study include the small number of patients, the lack of study design and of prespecified variable for multivariable analysis and the fact that tumor samples were not prospectively collected under controlled conditions, which can influence results of IHC. Hence, external validation and multivariable analyses including more variables such as those included in the nomogram may improve the confidence in the independent impact of p-EGFR [5]. Comprehensive tumor genomic analysis is also warranted. Detection of p-EGFR could suggest not only the need for adjuvant therapy, even in patients with N0 or N1 disease, but may potentially be predictive of benefit from the the use of anti-EGFR drugs. Indeed one case report has described the activity of panitumumab as second-line therapy in penile cancer, which exhibited amplified EGFR with no K-Ras mutations [19]. Of note, we recently reported that mutations commonly found in patients with non-small-cell lung cancer were not detected in penile cancer patients [20], which shows that the biological mechanism underlying EGFR activation remains to be fully explored.

## Conclusions

Our hypothesis-generating study showed that p-EGFR can be a powerful tool for prognosis discrimination of penile cancer patients with limited or no lymphnode involvement. Our study is the first to show such a relationship in a selected population not normally candidate for adjuvant therapy. The clinically relevant risk of disease recurrence strongly highlights the need for further stratification factors in such selected population of surgical PC patients. Our findings may have a huge impact as not only could they select N0-1 patients in need for adjuvant therapy, but could also select patients responsive to anti-EGFR therapy. This fascinating hypothesis requires external validation in a larger dataset, and integration of p-EGFR with classic prognostic factors and other histology markers should be pursued to establish optimal adjuvant therapy for these patients.

## Competing interests

The authors declare that they have no competing interests.

## Authors’ contribution

RF and GB performed the pathology analysis. RF, VG, GG, GB and RF carried out the immunohistochemistry studies. GP assessed the HPV status. CB performed the statistical analysis of the study and drafted the paper. SP, GDL, SDP, CB, PAA and RF conceived the study, and participated in its design and coordination. VG, MF, PR, DT, RDT collected clinical data. SP and GQ performed surgery. SP, GQ, PAA, RDT followed up patients after operation. GDL and GS gave inputs for study design and critically revised the paper. All authors read and approved the final manuscript.
